# Supplementary material for: Impact of Fecal Microbiota Transplant Formulations, Storage Conditions, and Duration on Bacterial Viability, Functionality, and Clinical Outcomes in Patients with Recurrent Clostridioides difficile Infection
Source: Microorganisms. 2025 Mar 4;13(3):587. doi: 10.3390/microorganisms13030587 (PMC11945259; doi:10.3390/microorganisms13030587)
Supplement: Supplementary file 1 [file microorganisms-13-00587-s001.zip › microorganisms-3487137-supplementary.pdf]

Impact of Fecal Microbiota Transplant Formulation, Storage Conditions, and Duration on Bacterial Viability, Functionality, and Clinical Outcomes in Recurrent *Clostridioides difficile* patients

Method S1: DNA extraction and 16S rDNA amplification and library preparation:

DNA was extracted from the live and dead cell fractions using PowerFecal Pro® DNA Isolation Kit from Qiagen following the manufacturer protocol using TissueLyser II at 25 Hz for 5 min for mechanical lysis of the cells. The 16S rRNA amplicon libraries were prepared with a two-step PCR amplification protocol targeting the V3-V4 variable region with the primers, 338F (5′- ACTCCTRCGGGAGGCAGCAG-3′) and 806R (5′-GGACTACHVGGGTWTCTAAT-3′) [26]. Step 1 PCR was performed with a target of 20 ng DNA as input, using KAPA HiFi HotStart ReadyMix (Roche CA) for 30 PCR cycles. ZymoBiomics microbial community DNA standard (Zymo Research) was included as library positive control and nuclease-free water as library negative control. Step 1 PCR primers contain a heterogeneity spacer that offsets the sequence reads by up to 7 bases and simultaneously increases multiplexing capacity. The second PCR step was carried out with 1 µl of DNA input (1:20 dilution of the step 1 PCR products) for 10 PCR cycles to barcode each sample with combinatorial dual indexes (CDI) using KAPA HiFi HotStart ReadyMix (Roche Canada). The quality of the amplification was evaluated with Invitrogen E-gel electrophoresis. Libraries were normalized and pooled with the SequalPrep™ Normalization Plate Kit. Library fragments of ~ 620 bp were selected with SPRI beads and the pooled library was sequenced on an Illumina MiSeq 600 cycle cartridge.

Method S2: SPME-GC×GC-TOFMS untargeted metabolomics:

First, 250 μL of the composite fermentation supernatant samples were mixed with 20 mg of NaCl (Sigma-Aldrich) and 50 μL of a 1 ppm aqueous solution of deuterated d5-Benzaldehyde (C/D/N Isotopes Inc., Canada) into 20-mL glass headspace vials (Chromatographic Specalties Inc., Canada). Headspace sampling by SPME was performed with a GERSTEL (GERSTEL Inc., Linthicum, MD, USA) multi-purpose auto-sampler using a Supelco® (Supelco®, Bellefonte, Pennsylvania, United States) Stableflex™ CVB/CAR/PDMS SPME fiber. Each sample underwent an incubation of 10 min at 40 °C, followed by an SPME extraction for 60 min at 40 °C. The samples were then introduced into the GC×GC system by desorption at 250 °C for 5 min. The 2D-chromatographic separation and identification of the metabolites were performed using a Leco BenchTOF (BT) 4D GC×GC-TOFMS (Leco Instruments, USA) with a cooled injection System (GERSTEL, USA). Ultra-pure helium (5.0 grade; Praxair Canada Inc., Canada) was used as the carrier gas, with a constant flow rate of 2.0 mL/min with a starting oven temperature of 40 °C, then to 180 °C at 5 °C/min and then 280 °C at 15 °C /min with a holding time of 1 min. Mass spectra were collected at an acquisition rate of 200 Hz over a mass range between 40 and 800 m/z across the entire chromatogram, with an electron impact energy of -70 eV. The samples were run in 5 batches, with instrument blanks, media blanks, alkanes, batch replicates, and a pooled QC for all samples in the batch.

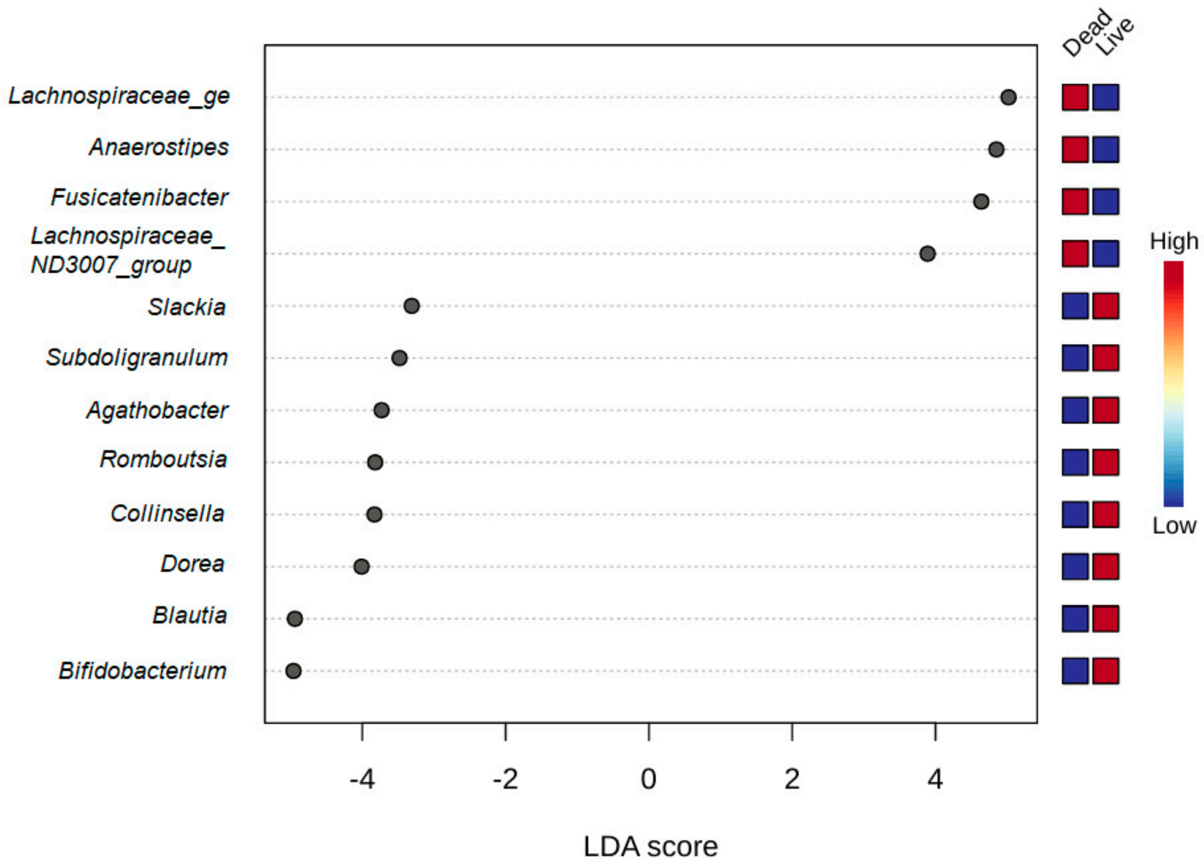

Figure S1. LEfSe analysis showing the statistically significant differentially abundant bacterial genera in live and dead cell fractions of FMT samples

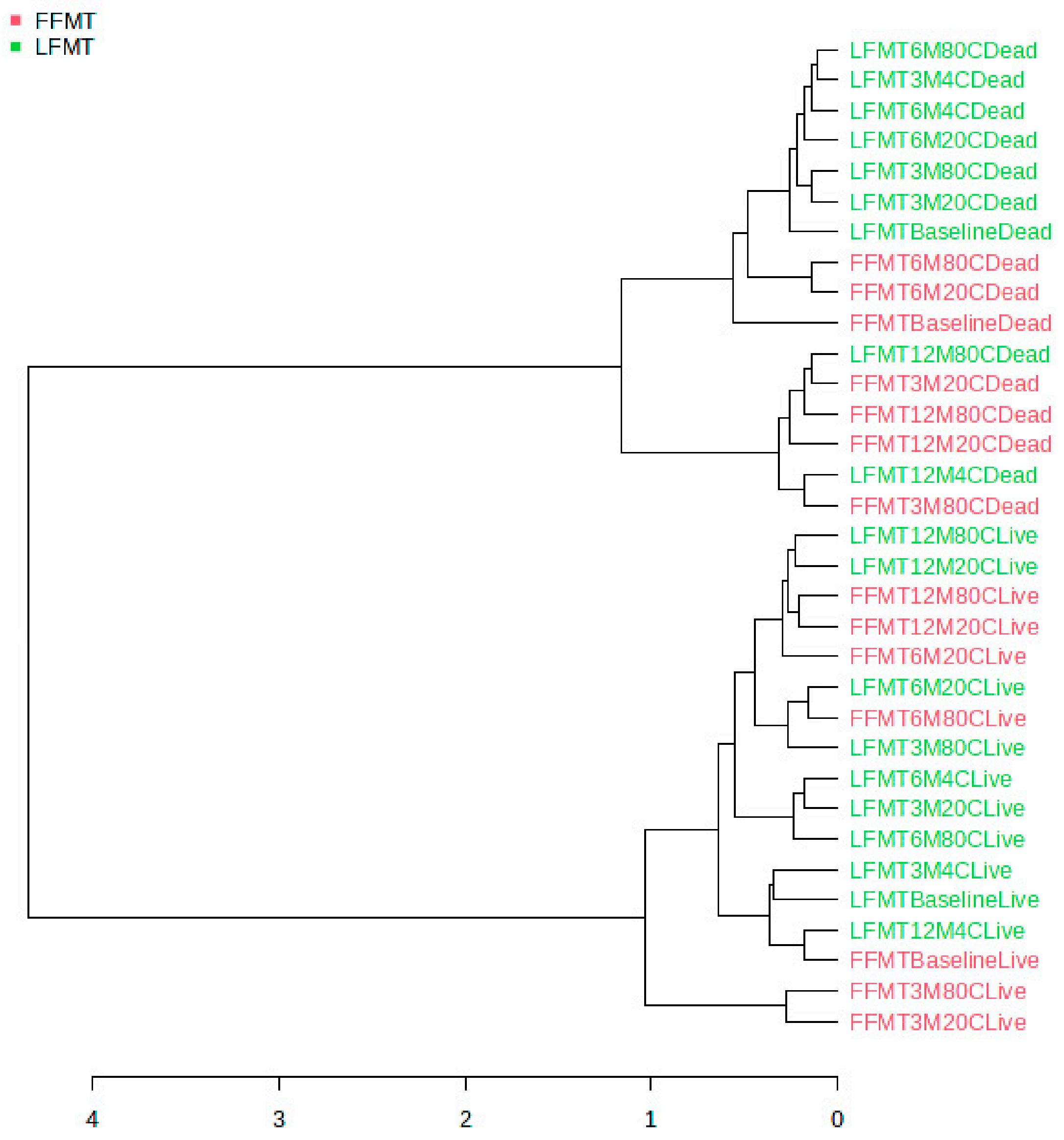

Figure S2. Clustering of the bacteria at the genera level separated the live and dead cell fractions of the FFMT and LFMT samples, indicating distinct microbial community features in these cell fractions and somewhat due to formulations.

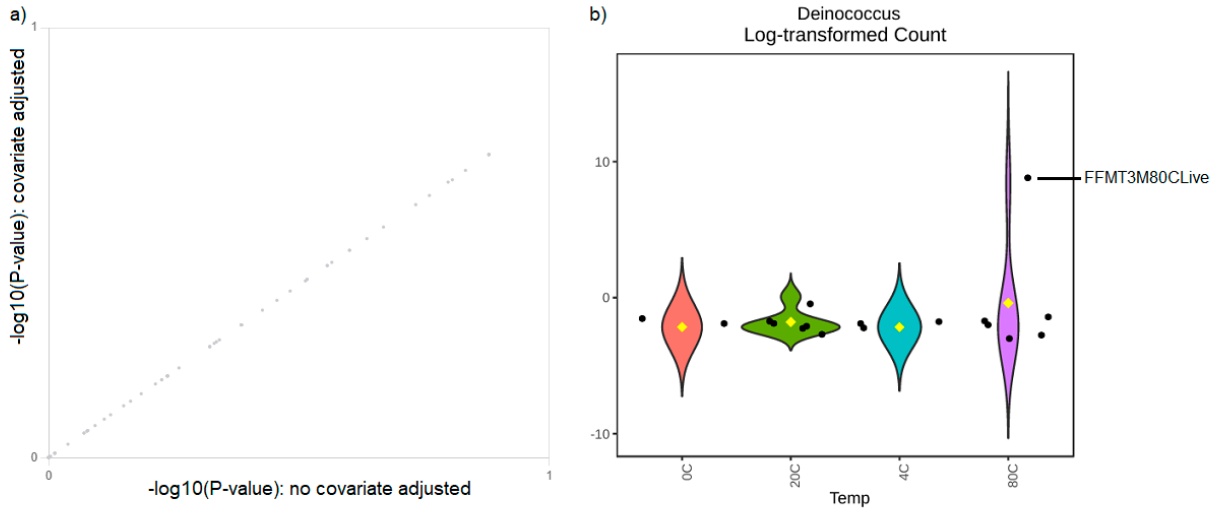

Figure S3. Effect of Storage Temperatures on the Bacterial Communities of Stored FMT (**a**) Multivariable regression analysis (MaAsLin2), both with and without adjusting for storage period as a covariate, revealed no statistically significant differences ($p > 0.05$) in bacterial community composition across different storage temperatures. (**b**) Univariate analysis (DESeq2) identified *Deinococcus* at the genus level as significantly more abundant ($p < 0.05$) in samples stored at -80°C compared to other storage temperatures. However, further examination revealed that this result was driven by the exceptionally high abundance of *Deinococcus* in a single sample (FFMT stored at -80°C for 3 months), suggesting that this finding may not reflect a broader trend across all samples.

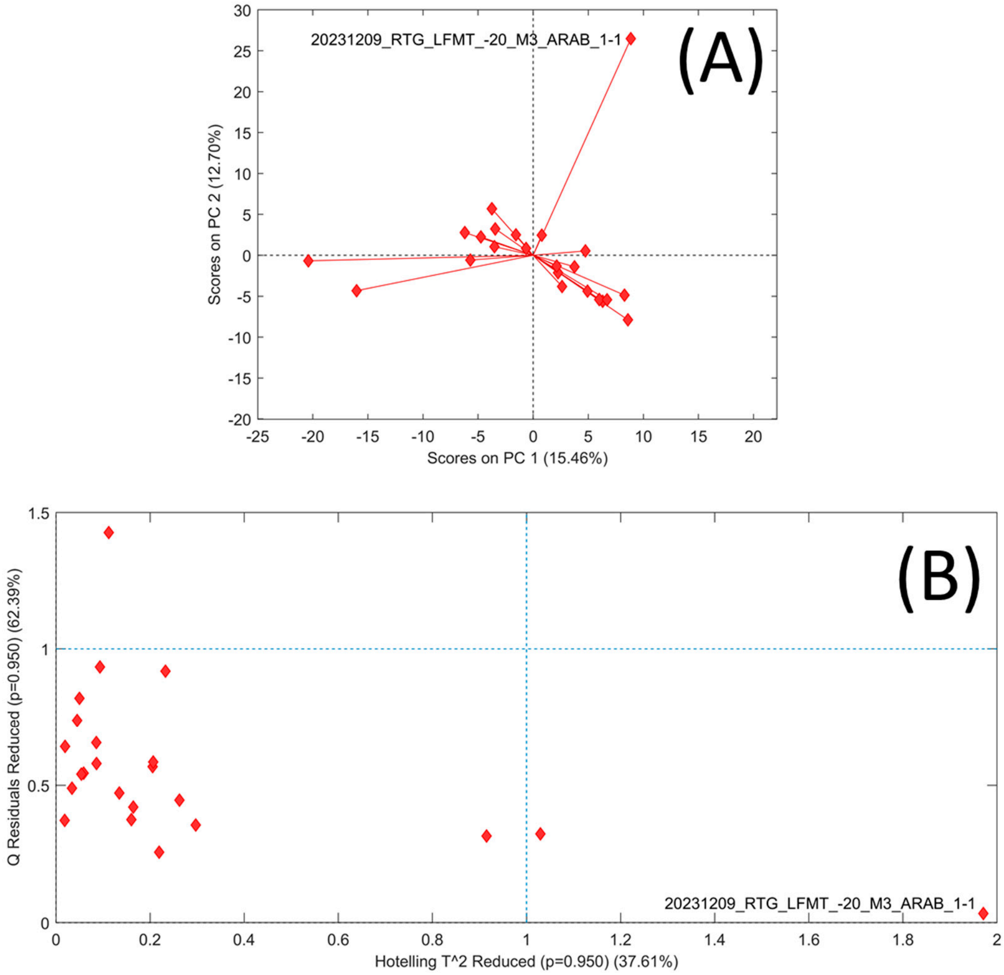

Figure S4. (**A**) PCA scores plot of all samples without class labels showing the suspected outlier (labeled), and (**B**) Q residual vs Hotelling T^2 plot showing the labeled suspected outlier with a high Hotelling T^2.

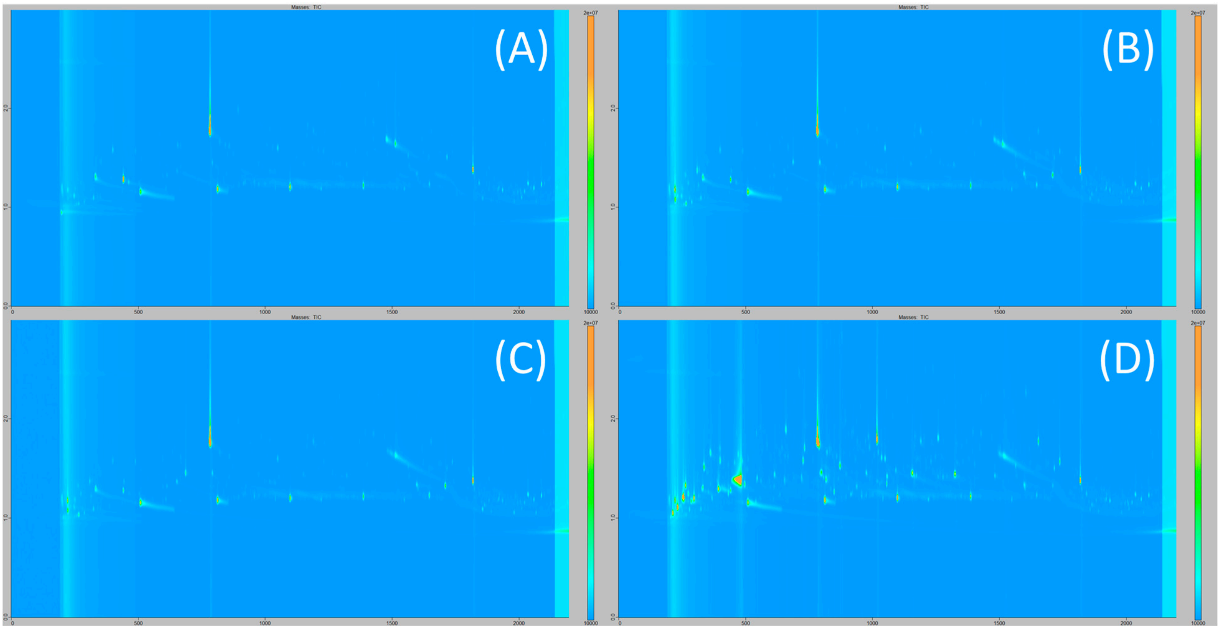

Figure S5. GC×GC-TOFMS total ion chromatograms (TIC) of blanks and a QC sample. The horizontal axis represents first-dimension retention times and the vertical axis represents second-dimension retention times. (**A**) reagent blank, water and sodium chloride, (**B**) arabinoxylan media blank, (**C**) inulin media blank, and (**D**) batch pool QC.

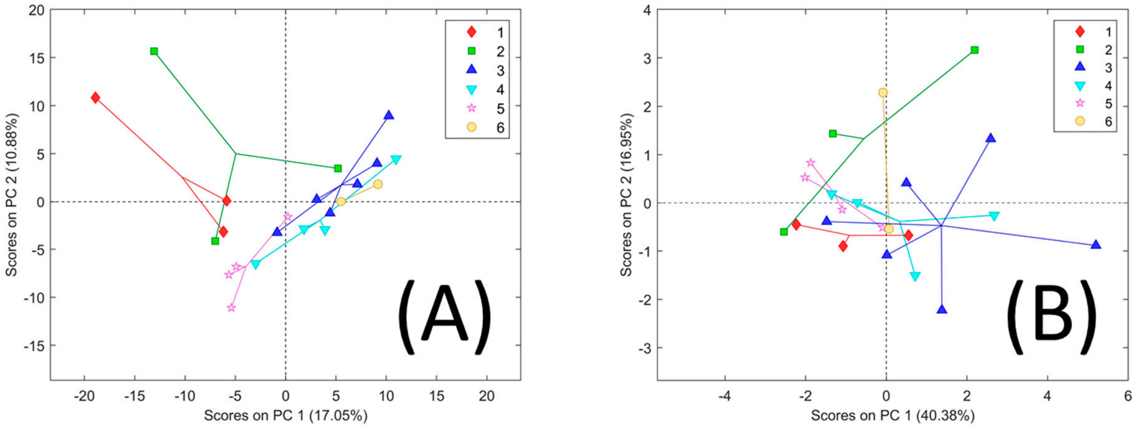

Figure S6. PCA scores plots with batch labels of (**A**) all variables and (**B**) just SCFAs, demonstrating minimal batch effects in the data. Separation of samples in batch 1 and 2 in (A) is the result of those batches containing samples which projected away based on their chemical characteristics rather than analytical batch effects alone (Figure S7). This is further reinforced by (**B**).

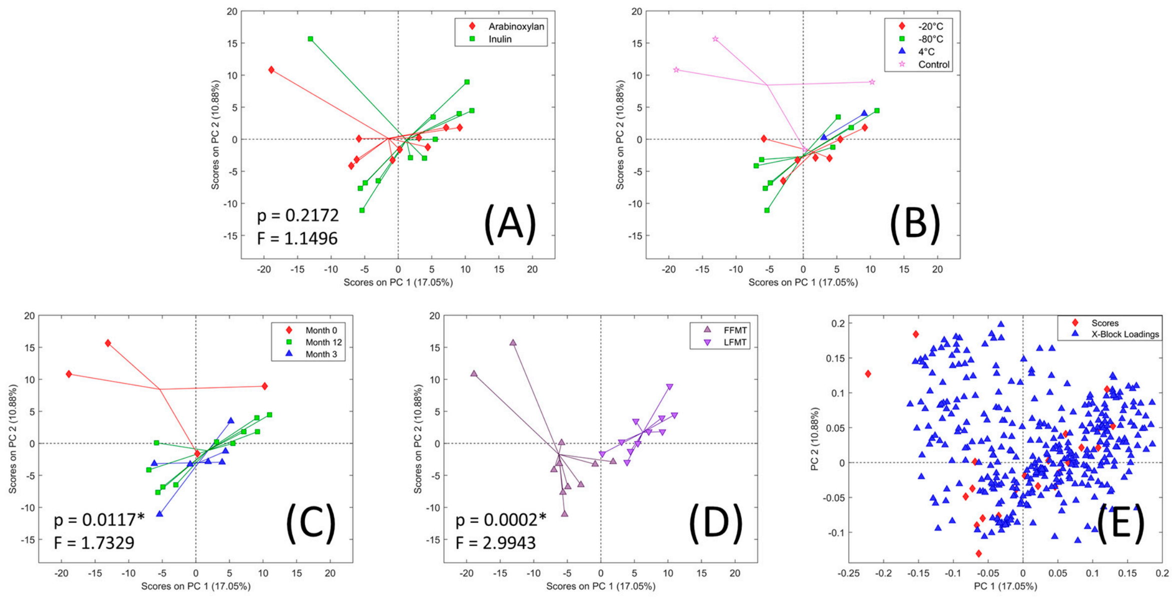

Figure S7. PCA scores plots generated considering all metabolites with p-value and F ratio from PERMANOVA on each scores plot, (**A**) Arabinoxylan vs. Inulin (**B**) temperatures, (**C**) time (month 0, month 3, and month 12), (**D**) FFMT vs LFMT and (**E**) biplot showing scores (red diamonds) and loadings (blue triangles). Scores represent individual samples and loadings represent individual variables and how they contribute to the principal components. * denotes statistical significance ($p < 0.05$). Summary statistics cannot be computed for B) due to low statistical power.

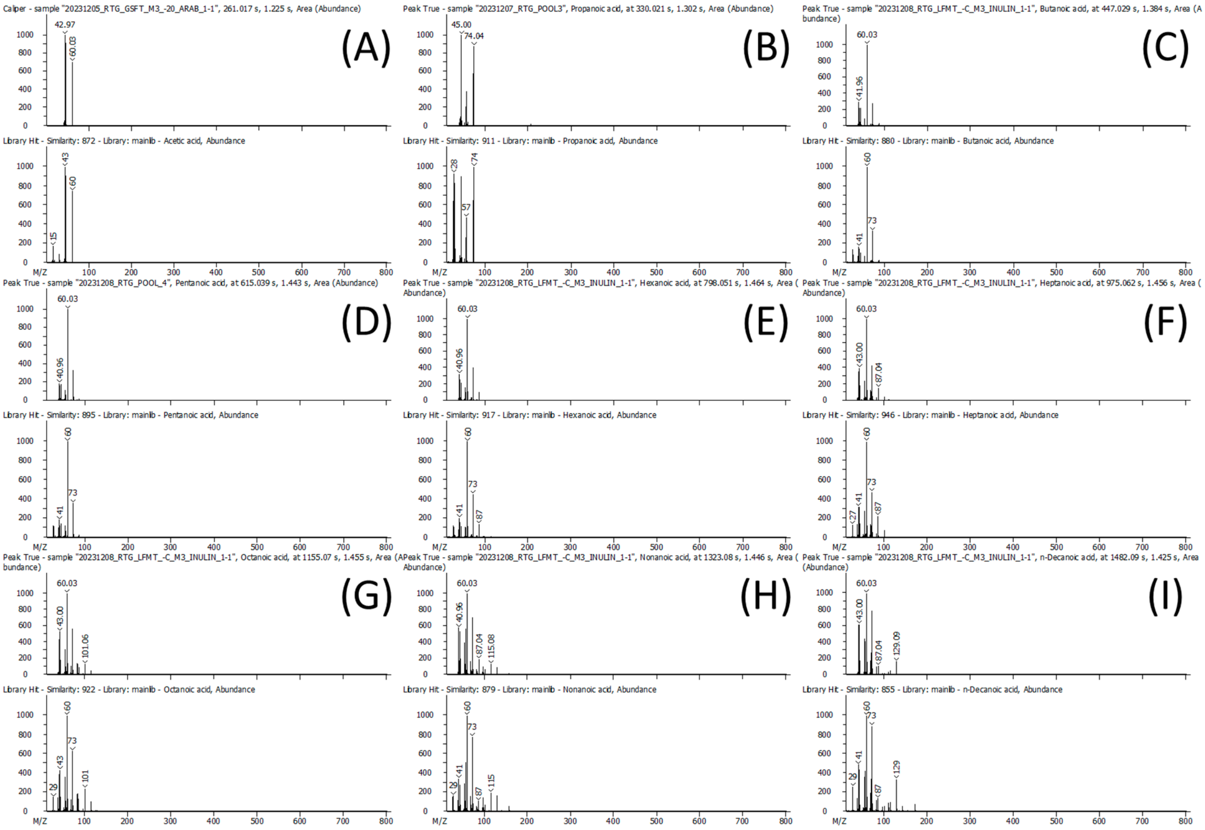

Figure S8. Experimental and library mass spectra for all SCFAs detected, (**A**) acetic acid, (**B**) propanoic acid, (**C**) butanoic acid, (**D**) pentanoic acid, (**E**) hexanoic acid, (**F**) heptanoic acid, (**G**) octanoic acid, (**H**) nonanoic acid, and (**I**) decanoic acid. Experimental mass spectra are on the top and library mass spectra are on the bottom for their respective metabolite.

Table S1. Results from PERMANOVA for all comparisons explored here, considering both all metabolites and only SCFAs.

**Comparison**	**F ratio**	**p-value**	**R^2**
All Metabolites FFMT vs LFMT	2.9943	0.0002	0.1302
All Metabolites Time	1.7329	0.0117	0.0797
All Metabolites Temperature	N/A	N/A	N/A
All Metabolites Fiber	1.1496	0.2172	0.0544
SCFAs FFMS vs LFMT	6.5008	0.00004	0.2453
SCFAs Time	1.0641	0.3626	0.0505
SCFAs Temperature	N/A	N/A	N/A
SCFAs Fiber	1.3873	0.2046	0.0649

Table S2. Library and experimental retention indices for the putatively identified SCFAs.

SCFA	Library RI	Experimental RI
Acetic acid	660	669
Propanoic acid	700	708
Butanoic acid	804	776
Pentanoic acid	902	872
Hexanoic acid	990	970
Heptanoic acid	1078	1065
Octanoic acid	1180	1163
Nonanoic acid	1273	1260
Decanoic acid	1373	1359
